# An Arylbenzofuran, Stilbene Dimers, and Prenylated Diels–Alder Adducts as Potent Diabetic Inhibitors from *Morus bombycis* Leaves

**DOI:** 10.3390/antiox12040837

**Published:** 2023-03-30

**Authors:** Seon Min Ju, Md Yousof Ali, Seung-Mi Ko, Jung-Hye Ryu, Jae-Sue Choi, Hyun-Ah Jung

**Affiliations:** 1 Department of Food Science and Human Nutrition, Jeonbuk National University, Jeonju 54896, Republic of Korea; 2 Department of Food Science and Nutrition, Pukyong National University, Busan 48547, Republic of Korea; 3 Department of Clinical Neurosciences, Hotchkiss Brain Institute, University of Calgary, Calgary, AB T2N4N1, Canada

**Keywords:** *Morus bombycis*, anti-diabetic, anti-diabetic complications, α-glucosidase, protein tyrosine phosphatase 1B, molecular docking simulation

## Abstract

*Morus bombycis* has a long history of usage as a treatment for metabolic diseases, especially, diabetes mellitus (DM). Thus, we aimed to isolate and evaluate bioactive constituents derived from *M. bombycis* leaves for the treatment of DM. According to bioassay-guided isolation by column chromatography, eight compounds were obtained from *M. bombycis* leaves: two phenolic compounds, *p*-coumaric acid (**1**) and chlorogenic acid methyl ester (**2**), one stilbene, oxyresveratrol (**3**), two stilbene dimers, macrourin B (**4**) and austrafuran C (**6**), one 2-arylbenzofuran, moracin M (**5**), and two Diels–Alder type adducts, mulberrofuran F (**7**) and chalcomoracin (**8**). Among the eight isolated compounds, the anti-DM activity of **3**–**8** (which possess chemotaxonomic significance in Morus species) was evaluated by inhibition of α-glucosidase, protein tyrosine phosphatase 1B (PTP1B), human recombinant aldose reductase (HRAR), and advanced glycation end-product (AGE) formation as well as by scavenging peroxynitrite (ONOO^−^), which are crucial therapeutic targets of DM and its complications. Compounds **4** and **6**–**8** significantly inhibited α-glucosidase, PTP1B, and HRAR enzymes with mixed-type and non-competitive-type inhibition modes. Furthermore, the four compounds had low negative binding energies in both enzymes according to molecular docking simulation, and compounds **3**–**8** exhibited strong antioxidant capacity by inhibiting AGE formation and ONOO^−^ scavenging. Overall results suggested that the most active stilbene-dimer-type compounds (**4** and **6**) along with Diels–Alder type adducts (**7** and **8**) could be promising therapeutic and preventive resources against DM and have the potential to be used as antioxidants, anti-diabetic agents, and anti-diabetic complication agents.

## 1. Introduction

Diabetes mellitus (DM) is one of the most serious health problems worldwide. According to the Diabetes Federation’s Global Diabetes Overview, there were 463 million people aged 20 to 79 years with diabetes in 2019, and this number is expected to continue to rise, reaching 700.2 million in 2045 [1]. Therefore, DM could cause hundreds of millions of individuals to experience serious health problems around the world in the future. DM, which is broadly divided into type 1 and type 2 DM (T2DM), is a metabolic disease caused by defects in insulin secretion and action [2]. In particular, dysfunction of α-glucosidase and protein tyrosine phosphatase 1B (PTP1B) was the main mechanism associated with T2DM [3]. The final stage of carbohydrate metabolism involves enzymatic breakdown into monosaccharides by α-glucosidase at the brush boundary of small intestine cells, and glucose uptake causes an increase in blood glucose [4]. Inhibiting carbohydrate digestion in the intestine is one method of controlling these carbohydrate-dependent diseases, and α-glucosidase inhibitors (AGIs) have been used to prevent DM, obesity, and hyperlipidemia by delaying carbohydrate digestion and absorption [5]. Protein tyrosine phosphatases (PTPs) are a large protein family that regulates a variety of physiological and pathological events. The endoplasmic reticulum of many tissues (including the liver, muscle, and fat) contains the enzyme PTP1B and is involved in insulin signaling. PTP1B has been identified as a key negative regulator for insulin and leptin signaling. Thus, inhibitors to decrease PTP1B levels augment insulin action [6,7,8].

Diabetic complications due to persistent hyperglycemia have been reported, including eye disease, retinopathy, nephropathy, and neuropathy [9]. Various hyperglycemia-linked pathways include increased polyol pathway flux, advanced glycation end-products (AGEs) formation, and oxidative stress [10]. Glucose, lipids, and/or specific amino acids on proteins and nucleic acids are non-enzymatically reduced to form AGEs. AGEs are associated with the generation of free radicals and oxidants [11]. The other is the polyol pathway, which consists of two enzymatic reactions. Glucose was reduced to sorbitol by aldose reductase (AR) with co-factor NADPH, and then sorbitol was converted to fructose by sorbitol dehydrogenase (SDH) with co-factor NAD (SDH) [12]. In hyperglycemia, the glutathione level is reduced through NADPH reduction when glucose is converted to sorbitol, and NAD^+^ is converted to NADH in the second enzymatic reaction to generate superoxide anions [13]. The reaction via the polyol pathway induces ROS production and generates oxidative stress. Reactive oxygen species (ROS) produced by cells in various enzymatic and non-enzymatic processes contain radicals, such as superoxide (O_2_^−^), hydroxyl (OH), peroxyl (ROO), hydroperoxyl (HOO), and non-radicals [14]. When non-reactive radicals and ROS such as superoxide react with nitric oxide (NO) produced in biological tissues, a reactive intermediate was formed to affect the function of proteins and the whole organism [15]. This reaction generates the peroxynitrite (ONOO^−^), which causes damage to a variety of tissues and organs, leading to energy depletion and cell necrosis. Moreover, ONOO^−^ facilitates the development of DM-related retinopathy, neuropathy, nephropathy, and cardiovascular problems [16].

Since hyperglycemia and insulin resistance are characteristics of T2DM, therapeutic agents or inhibitors that reduce postprandial hyperglycemia and hyperinsulinemia and improve insulin sensitivity have been used to treat T2DM [4,5,8]. However, synthetic drugs, including acarbose and voglibose (used in the treatment of T2DM), have been linked to gastrointestinal side effects, such as abdominal pain, bloating and increased frequency, and severity of stools that can lead to bacterial fermentation due to undigested carbohydrates [17]. The toxicity report says that pharmaceuticals made from natural products have fewer or no side effects as compared to synthetic drugs [5]. As a result, it is becoming more important to conduct research on natural-product-based T2DM inhibition.

*Morus*, a plant genus of the Moraceae family, is well known for its distribution in northeast Asia, such as China, Japan, and Korea. *Morus* genus is composed of approximately 16 species [18], and some of them (e.g., *Morus mongolica*, *M. nigra*, *M. lhou*, and *M. alba*) have been used as valuable traditional medicines and essential feed for silkworms [19]. In particular, *Morus* species were verified to have bioactive substances, such as arylbenzofurans [20], chalcone-derived Diels–Alder adducts [21], and flavonoids [22]. These bioactive substances are generally expected to have their own medical efficacy with antioxidant [20], anti-diabetic [23], and anti-Alzheimer’s disease properties [24]. Mulberrofuran G, a representative Diels–Alder type isolated from *Morus* species, has been reported as a potent inhibitor against ROS generation [25], tyrosinase [26], and PTP1B [27]. While each of the 16 kinds of *Morus* species has different medical effects, we focused on three dominant and widely cultivated species, including *M. alba, M. lhou*, and *M. bombycis*, to compare the medical efficacy. *Morus alba*, called *Baek-sang* in Korean, has bioactive substances, such as mulberroside F, chalcomoracin, oxyresveratrol, moracin derivatives, flavonoids, and flavonoid glycoside derivatives, which engage in biological activities such as anti-diabetic and anti-tyrosinase effects [28]. *Morus lhou*, called *No-sang* in Korean, has been reported to have bioactive compounds inhibiting β-secretase, tyrosinase, and cholinesterase. In addition, this plant is reported to harbor prenylated flavonoids (morusin, morusinol, and flavones), flavones (norartocarpetin and kuwanon C), and phenolic compounds (mulberrosides A and C), exhibiting these bioactivities [29,30]. *Morus bombycis*, called *San-sang* in Korean, is a wild-type plant from the mountains. Although its appearance is similar to that of *M. alba*, its fruit is smaller, and its pistil is divided into two parts, the stigma and ovary. *Morus bombycis* is reported to exhibit anti-inflammatory [31], anti-diabetic [32,33], anti-obesity [34], skin-whitening [35], and anti-Alzheimer’s disease effects [36]. Phytochemical studies on *M. bombycis* have demonstrated the presence of moracinoside M, mulberrofuran K, kuwanon V, oxyresveratrol, such as Diels–Alder type adducts, moracin glycoside derivatives, flavone, flavonoid glycoside derivatives, and chalcone derivatives [31,37]. In particular, 1-deoxynojirimycin (1-DNJ) and *N*-methyl-1–DNJ have been found in the leaves of *M. bombycis*, and they have been shown to have a strong anti-diabetic effect [38].

Although *Morus* species were traditionally used as medicines due to their pharmacological properties, there are limited studies on the bioactivity of *M. bombycis* leaves and their pharmacological compounds. Thus, a more detailed physiological action and phytochemical analysis of *M. bombycis* were performed in this study. In particular, two stilbene dimers and two Diels–Alder type adducts were isolated from *M. bombycis* leaves at first along with four compounds. We investigated the anti-diabetic and antioxidant properties of *M. bombycis* and its major constituents as part of our ongoing efforts to identify potent inhibitors against PTP1B, α-glucosidase, AGEs formation, and antioxidant agents from natural sources. The mode of inhibition or molecular interactions of active compounds with corresponding enzymes such as PTP1B and α-glucosidase were investigated. Furthermore, various in vitro anti-diabetic complication assays were used to evaluate the inhibitory effects of active compounds on AGE formation and HRAR. Overall, we sought to substantiate the anti-diabetic, anti-diabetic complications, and antioxidant effects of the compounds isolated from *M. bombycis* leaves.

## 2. Materials and Methods

### 2.1. General Experimental Procedures

The ^1^H and ^13^C NMR spectra were recorded using a JEOL spectrometer (JNM-ECZ500R, Tokyo, Japan) at 500 and 125 MHz for ^1^H NMR and ^13^C NMR in deuterated solvent (methanol-*d*_4_ (CD_2_OD), acetone-*d*_6_ ((CD_3_)_2_CO)). Various column chromatography methods were implemented using a silica (Si) gel 60 (70–230 mesh, Merck, Darmstadt, Germany), Lichroprep^®^ RP-18 (40–63 µm, Merck, Darmstadt, Germany), and Sephadex LH-20 (20–100 μM, Sigma, St. Louis, MO, USA). The ultra-performance liquid chromatography (UPLC) was analyzed using a UPLC-diode array detector (DAD) (Water Co., Milford, MA, USA) and a quadrupole time of flight mass spectrometry (QToF/MS) (Waters Micromass, Manchester, UK) equipped with CORTECS UPLC T3 column (150 × 2.1 mm^2^ i.d., Phenomenex, Torrance, CA, USA). Thin-layer chromatography (TLC) was performed on pre-coated Merck Kieselgel 60 F_254_ plates (20 × 20 cm^2^, 0.255 mm, Merck) and RP-18 F_254_S plates (5 × 10 cm^2^, Merck), using 10% H_2_SO_4_ (sulfuric acid dissolved in methanol) as a spray reagent. All solvents for column chromatography were reagent grade and were purchased from commercial sources.

### 2.2. Chemicals and Reagents

1,1-Diphenyl-2-picrylhydrazyl (DPPH), 2,2′-azinobis(3-ethylbenzothiazolin-6-sulfonic acid) diammonium salt (ABTS), L-ascorbic acid, 6-hydroxy-2,5,7,8-tetramethylchloman-2-carboxylic acid (Trolox), acarbose, bovine serum albumin (BSA), human serum albumin (HSA) aminoguanidine hydrochloride, diethylene triamine pentaacetic acid (DTPA), ethylene diamine tetraacetic acid (EDTA), DL-dithiothreiol (DTT), dihydrorhodamine 123 (DHR123), D-(-)-fructose, D-(+)-glucose, *p*-nitrophenyl α-D-glucopyranoside (*p*NPG), *p*-nitrophenyl phosphate (*p*NPP), β-nicotinamide adenine dinucleotide 2′-phosphate reduced tetrasodium salt hydrate (NADPH), and α-glucosidase from *Saccharomyces cerevisiae* were purchased from Sigma-Aldrich Chemical Company (St. Louis, MO, USA). Protein tyrosine phosphatase 1B (PTP1B) was purchased from ENZO (Farmingdale, NY, USA). Human recombinant AR (0.4 units) was purchased from Wako Chemicals (Osaka, Japan). Citric acid trisodium salt dehydrate, citric acid monohydrate, sodium carbonate anhydrous, sodium chloride, sodium phosphate dibasic anhydrous, and sodium phosphate monobasic dehydrate were purchased from SAMCHUN (Seoul, Korea). Peroxynitrite (ONOO^−^), 3-[(3-cholamidopropyl)dimethylammonio]-1-propanesulfonate (CHAPS), and sodium azide were purchased from Bio-Rad Laboratories Calbiochem (San Diego, CA, USA), GENERAY BIOTECH (Shanghai, China), and JUNSEI (Chuoku, Tokyo, Japan), respectively.

### 2.3. Plant Material

The leaves of *M. bombycis* were collected at Jeju in September 2021 and purchased from JEJU SAN YA CHO (Jeju, Korea). A voucher specimen as leaves is registered (MB202109002) and deposited at the Department of Food Science and Human Nutrition, Jeonbuk National University, Jeonju, South Korea (Professor H. A. Jung).

### 2.4. Extraction, Fractionation, and Isolation

**Figure 1:**
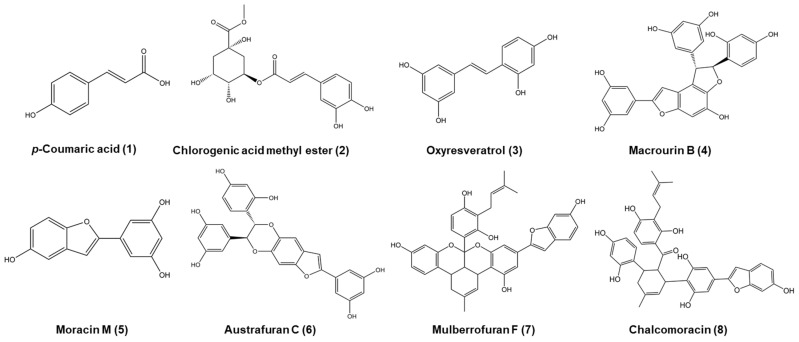
The structures of isolated compounds from *Morus bombycis* leaves.

The air-dried leaves of *M. bombycis* (18 kg) were properly crushed, extracted, and refluxed with hot methanol (MeOH) for 3 h (6 L × 5 times). After filtering the extract, the solvent of the total filtrate was removed from the rotary evaporator at 80 °C or reduced pressure to acquire the crude MeOH extract (2.05 kg). This extract was suspended in a funnel in distilled water (H_2_O) and partitioned sequentially with methylene chloride (CH_2_Cl_2_), ethyl acetate (EtOAc), and *n*-butanol (*n*-BuOH) to yield CH_2_Cl_2_ (660.98 g, 32.10%), EtOAc (66.38 g, 3.22%), *n*-BuOH (284.31 g, 13.81%), as well as H_2_O residue (1035.3 g, 50.27%), respectively. The EtOAc fraction (66.38 g) was first separated using column chromatography on a Sephadex LH-20 using an isocratic solvent of MeOH to obtain 8 subfractions (E1 to E8). The fraction of E3 was separated by silica gel column chromatography, eluting with a gradient mixture solvent of CH_2_Cl_2_:MeOH (40:1, gradient) to yield 12 fractions (E3-1 to E3-12). The fraction of E3-6 was separated by reverse phase-18 (RP-18) (H_2_O:MeOH, 1:0, gradient) to yield 6 fractions (E3-6-1 to E3-6-6) and give compound **1** (15 mg) and compound **2** (11 mg). Compound **1** was identified as *p*-coumaric acid [39]. Compound **2** was identified as chlorogenic acid methyl ester [40]. The fraction E5 was separated by silica gel column chromatography, eluting with a gradient mixture solvent of CH_2_Cl_2_:MeOH (40:1, gradient) to yield 12 fractions (E5-1 to E5-12). The fraction E5-5 was separated by repeated silica gel column chromatography, eluting with an isocratic mixture solvent of *n*-hexane:EtOAc:MeOH (5:3:0.5, gradient) to yield 5 fractions (E5-5-1 to E5-5-5). Compound **3** (141.7 mg) was isolated from the fraction of E5-5-2 separated by RP-18 (H_2_O:MeOH, 4:6, gradient) and purified by RP-18 under the same conditions. Compound **3** was identified as oxyresveratrol [41]. The fraction E4 was separated by silica gel column chromatography, eluting with a gradient mixture solvent of CH_2_Cl_2_:MeOH (40:1, gradient) to yield 7 fractions (E4-1 to E4-7). The fractions E4-2 and E4-3 were separated by RP-18 (H_2_O:MeOH, 3:7, gradient) to give compound **5** (5.2 mg) and yield 5 fractions (E4-3-1 to E4-3-5), respectively. The fraction E4-3-3 was separated by repeated RP-18 (H_2_O:MeOH, 7:3, gradient) to give compound **5** (16.8 mg). The fraction E5-4 was separated by RP-18 (H_2_O:MeOH, 8:2, gradient) to yield 5 fractions (E5-4-1 to E5-4-5). The fraction E5-4-2 was separated by Sephadex LH-20 with an isocratic solvent of MeOH to give compound **5** (37.5 mg). Compound **5** was identified as moracin M [42]. The fraction E8 was separated by RP-18 (H_2_O:MeOH, 7:2, gradient) to yield 4 fractions (E8-1 to E8-4). The fraction E8-2 was purified by RP-18 (H_2_O:MeOH, 6:4, gradient) to give compound **4** (137.9 mg). Compound **4** was identified as macrourin B [43]. The fraction E8-4 was separated by silica gel column chromatography with a gradient mixture solvent of CH_2_Cl_2_:MeOH (40:1, gradient) to yield 7 fractions (E8-4-1 to E8-4-7). The fraction E8-4-6 was repeatedly separated by RP-18 (H_2_O:MeOH, 4:6, gradient) to give compound **6** (18.9 mg). The fraction E8-4-2 was separated by RP-18 (H_2_O:MeOH, 4:6, gradient) to give compound **7** (11.9 mg). Compound **6** was identified as austrafuran C [44], and compound **7** was identified as mulberrofuran F [45]. The fraction E8-4-5 was separated by RP-18 (H_2_O:MeOH, 1:1, gradient) to yield 7 fractions (E8-4-5-1 to E8-4-5-7). The fraction E8-4-5-7 was separated by RP-18 (H_2_O:MeOH, 4:6, gradient) to yield 4 fractions (E8-4-5-7-1 to E8-4-5-7-4). The fraction E8-4-5-7-4 was purified by RP-18 (H_2_O:MeOH, 4:6, gradient) to give compound **8** (46.1 mg). Compound **8** was identified as chalcomoracin [46]. The identities of all isolated compounds were determined based on spectroscopic analyses, including ^1^H NMR, ^13^C NMR, HMBC, HMQC, DEPT, COSY, and NOESY (Tables S2–S4; Figures S1 and S2). These data were compared to the spectroscopic data reported in previous literature. The structures of isolated compounds are shown in Figure 1.

### 2.5. UPLC-QToF/ESI-MS Analysis

UPLC-QToF/ESI-MS analysis was performed to identify and quantify components from the methanol extract and its organic solvent fractions from *M. bombycis* leaves. According to the operating protocol [47], LC chromatogram and mass spectra were simultaneously measured. Briefly, 1 µL of the sample (2 µg/µL) was injected into column (30 °C) and run for 40 min at a flow rate of 0.35 mL/min. Solvent system consists of mobile phase A (0.5% formic acid in water) and B (0.5% formic acid in acetonitrile), eluting gradient condition. Mass spectra were operated within the range of *m*/*z* 50–800 in positive ionized mode using a positive ESI probe, and their parameters were capillary voltage 3.5 kV, sampling cone voltage 40 V, source temperature 120 °C, desolvation temperature 400 °C, and desolvation N_2_ gas flow 1000 L/h.

### 2.6. Determination of Total Phenolic Content (TPC) and Total Flavonoids Content (TFC)

TPC and TFC measurements of the extract and each fraction obtained from *M. bombycis* leaves were conducted according to previous literature with some modifications [48].

### 2.7. Assay for Scavenging Activity against ABTS Radical and DPPH Radical

The ABTS and DPPH radical scavenging activity of the extract and each fraction obtained from *M. bombycis* leaves were measured according to previous literature with modifications [48].

### 2.8. In Vitro Assay for ONOO^−^ Scavenging Activity

ONOO^−^ scavenging activity was measured using the method in previous literature involving measuring highly fluorescent rhodamine 123 that is converted from non-fluorescent DHR123 in the presence of ONOO^−^ [49].

### 2.9. In Vitro Assay for Inhibitory Activity of α-Glucosidase and PTP1B Enzyme

The enzyme inhibition study was executed spectrophotometrically following the previous literature [49]. Acarbose and ursolic acid were used as the positive controls for α-glucosidase and PTP1B, respectively.

### 2.10. Kinetic Parameters of Isolated Compounds for Inhibition of α-Glucosidase and PTP1B Using Lineweaver–Burk and Dixon Plots

The two kinetic methods, Lineweaver–Burk plots and Dixon plots, were used to determine the inhibition mechanism [49,50,51,52]. The α-glucosidase inhibition type was measured at various concentrations of substrate (*p*NPG, 0.625, 1.25, and 2.5 mM) and several concentrations of test compounds (0.8, 0.4, and 0.16 μM for compound **4**; 2, 0.8, and 0.16 μM for **6**; 3.17, 1.59, and 0.63 μM for **7**; 1.54, 1.04, and 0.62 μM for **8**) using Lineweaver–Burk double reciprocal plots. The PTP1B enzyme inhibition type was also measured at various concentrations of substrate (*p*NPP, 0.5, 1.0, 2.0, and 4.0 mM) and several concentrations of test compounds (4.0, 2.0, and 0.8 μM for compound **4**; 2.0, 0.8, and 0.16 μM for **6**; 15.87, 7.93, 3.17, and 0.63 μM for **7**; 6.17, 3.08, 1.54 and 0.78 μM for **8**) using Lineweaver–Burk double reciprocal plots. Dixon plots were used to determine the inhibition constant (*K*_i_) of each compound by testing the effects of substrate and compounds against the α-glucosidase and PTP1B under the same conditions as described above.

### 2.11. In Silico Molecular Docking Analysis for α-Glucosidase and PTP1B Inhibition

Before the docking analysis to investigate the binding poses of compounds inside the active receptor pockets, the crystal protein structures for PTP1B (PDB ID: 1NNY for the catalytic site; 1T49 for the allosteric site) and α-glucosidase (PDB ID: 3A4A) were downloaded from the Protein Data Bank (PDB) [53]. These protein structures were confirmed using X-ray diffraction. The reported heteroatom compounds and water molecules were removed, and the protein was regarded as ligand-free for the docking simulation using Accelrys Discovery Studio 19.1 (http://www.accelrys.com, accessed on 1 January 2023; Accelrys Inc., San Diego, CA, USA). Polar hydrogen atoms were added to the protein using an automated docking tool, AutoDock 4.2.6. [54]. The docking studies for macrourin B (**4**), austrafuran C (**6**), chalcomoracin (**7**), mulberrofuran F (**8**), acarbose, and co-crystalline ligands were performed without modifying the default parameters. The 2D structures of all the compounds were drawn with MarvinSketch (www.chemaxon.com, accessed date 1 January 2023; Chemaxon, Life Science, Informatics, Cheminformatics, Budapest, Hungary); Chemaxon, Budapest, Hungary). Energy minimization of each ligand was carried out using the molecular mechanics 2 (MM2) force field, and the docking analysis was conducted using AutoDock Vina [55]. A grid box size of 60 × 60 × 60 points with a spacing of 1.0 Å between the grid points was executed to cover almost all the favorable protein-binding sites. The X, Y, Z centers were PTP1B (56.019, 31.36, and 22.48), and α-glucosidase (21.28, −0.75, and 18.63). In the docking studies, the selected ligands (all compounds) were examined to find qualified binding poses for each compound. The binding aspects of the PTP1B and α-glucosidase residues and their corresponding binding affinity scores are regarded as the best molecular interactions.

### 2.12. In Vitro Assay for Inhibitory Activity of HRAR and AGEs Formation

The inhibitory activity of HRAR was examined according to previous literature with modifications [56]. First, 150 µL of 100 mM sodium phosphate buffer (pH 6.2), 20 µL of 0.3 mM NADPH as the co-enzyme, 5 µL of the test samples (50, 10, and 2 mg/mL or 100% DMSO), and 20 µL of 10 mM DL-glyceraldehyde as the substrate were added to each of the 96 wells (final volume 200 µL). Quercetin was used as a positive control. The inhibitory activity of AGEs formation was examined according to the modified method [49]. Aminoguanidine hydrochloride was used as a positive control for the AGEs formation inhibition assay.

### 2.13. Statistics

All results are expressed as the mean ± SD of triplicate experiments. Statistically significant differences were determined by analysis of variance (ANOVA) and Duncan’s test (Systat Inc., Evanston, IL, USA). A *p*-value < 0.05 was considered statistically significant.

## 3. Results

### 3.1. Phytochemical and Bioactivity Analysis of Morus Species

#### 3.1.1. Preliminary Experiment of Three Dominant Morus Species

In the preliminary experiments of three dominant and widely cultivated species, including *M. alba*, *M. lhou*, and *M. bombycis*, the MeOH extract of the last species exhibited α-glucosidase inhibitory activity and a higher TPC value, while the MeOH extracts of the first two species showed good antioxidant capacity and higher content in total flavonoids (Table 1). Therefore, the leaves of *M. bombycis* were selected as promising candidates for anti-diabetic therapy, and further research on the evaluation of anti-diabetic activity and phytochemical analysis was performed.

#### 3.1.2. Phytochemical Analysis of the *Morus bombycis* Leaves

MeOH extract and its fractions were obtained by successively partitioning the MeOH extract with several organic solvents, and their antioxidant and anti-diabetic activities were investigated. The EtOAc fraction was first separated by Sephadex LH-20 to obtain eight fractions (E1−E8) based on bioactivity-guided fractionation. The E3 fraction had the strongest inhibitory activity in antioxidant activity through ONOO^−^ scavenging, while E6 and E8 fractions showed significant inhibitory activity via α-glucosidase, PTP1B, and AGEs (Table S1) in the anti-diabetic and anti-diabetic complication study. According to bioassay-guided fractionation, E3 to E8 fractions were individually separated by column chromatography to obtain the eight compounds shown in Figure 1. These chemical structures were determined based on analysis of 1H NMR, 13C NMR, DEPT, HMQC, HMBC, COSY, and NOESY. The known compounds were confirmed by the spectral data of previous literature (Tables S2–S4; Figures S1 and S2). Macrourin B (**4**), isolated as a brown amorphous powder, was found to have the molecular formula C_28_H_20_O_9_ using ESI-MS (*m*/*z* 501.01 [M + H]^+^, calculated for C_28_H_20_O_9_) with its 1D and 2D NMR data [43]. The 1H NMR spectroscopic data of **4** showed two sets of signals for 3,5 dihydroxybenzene moieties (δ_H_ 6.65 (2H, *J* = 2 Hz), 6.23 (2H, d, *J* = 2.5 Hz) 6.19 (1H, t, *J* = 3.2 Hz), 6.17 (1H, t, *J* = 4.6 Hz)); one set of signals for 2,4 dihydroxybenzene moieties (δ_H_ 7.10 (1H, d, *J* = 8.5 Hz), 6.33 (1H, d, *J* = 2.5 Hz), 6.27 (1H, dd, *J* = 8.4 Hz, 2.3 Hz)); one aromatic proton (δ_H_ 6.90 (1H, s)), one proton of furan ring (δ_H_ 6.43 (1H, br s)); two coupled doublets (4.74 (1H, d, *J* = 7 Hz), 5.78 (1H, d, *J* = 7.5 Hz)). The ^1^H-^1^H COSY indicated correlation of H-5′/6′; H-7′/8′; and H-9/11. In the HMBC spectrum of **4**, the CH long-range correlation showed between H-3/C-(4, 3a, 5, 8); H-7/C-(4, 6, 7a, 5); H-11/C-(9, 10, 12); H-13/C-(8, 9, 12); H-3′/C-(1′, 2′, 4′ 5′); H-5′/C-(1′, 2′, 3′, 4′); H-7′/C-(3a, 1′, 2′, 6′, 8′, 9′); H-8′/C-(3a, 1′, 7′, 9′ 10′); H-10′, 14′/C-(8′, 12′, 13′ 14′); H-12′/C-(9′, 11′, 13′). The NOE correlation between H-7′ and H-10′ (14ʹ), as well as between H-8′ and H-6′, indicated a *trans* orientation of H-7′ and H-8′. Austrafuran C (**6**), a brown amorphous powder, was very similar to the 1D NMR data of macrourin B (**4**), and it had the same molecular formula C_28_H_20_O_9_ [44]. A previous study demonstrated the reasons for the similarity between the 1D NMR spectra of mulberrofuran F (**7**) and chalcomoracin (**8**) [45,46].

**Figure 2:**
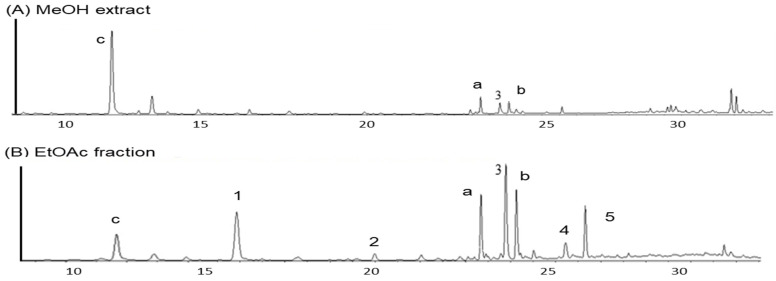
UPLC chromatograms of MeOH extract (**A**) and EtOAc fraction (**B**). Peak 3 (chlorogenic acid); Peak 1 (*p*-coumaric acid): 12.83 min; Peak 2 (chlorogenic acid methyl ester): 19.95 min; Peak a (cuercetin 3-*O*-ß-D-glucoside): 20.55 min; Peak 3 (cxyresveratrol): 22.85 min; Peak b (kaempferol 3-*O*-ß-D-glucoside): 23.40 min; Peak 4 (macrourin B): 25.18 min; Peak 5 (moracin M): 25.80 min.

UPLC analysis was conducted to qualitatively estimate the active components in the EtOAc fraction. As shown in Figure 2, the peaks of seven constituents of the EtOAc fraction were confirmed by UPLC qualitative analysis. The UPLC profiles showed the presence of kaempferol-3-*O*-ß-D-glucoside (a) and quercetin-3-*O*-ß-D-glucoside (b) as two major flavonoids, and oxyresveratrol (**3**) as one major stilbene, as well as *p*-coumaric acid (**1**), chlorogenic acid methyl ester (**2**), macrourin B (**4**), and moracin M (**5**) as minor constituents. The retention times of compounds in the EtOAc fraction are as follows: *p*-coumaric acid (**1**, 12.83 min), chlorogenic acid methyl ester (**2**, 19.95 min), quercetin 3-*O*-ß-D-glucoside (20.55 min), oxyresveratrol (**3**, 22.85 min), kaempferol 3-*O*-ß-D-glucoside (23.40 min), macrourin B (**4**, 25.18 min), and moracin M (**5**, 25.80 min).

Along with the UPLC analysis, the total phenol content and total flavonoid content of the MeOH extract of *M. bombycis* leaves and its organic solvent fractions were also determined to assess the content of bioactive components (Table 2). According to the results of TPC, the EtOAc fraction of *M. bombycis* leaves showed the highest TPC value. The other three fractions showed high TPC values in the order of *n*-BuOH, CH_2_Cl_2_, and H_2_O residue. Likewise, in the TFC value, the EtOAc fraction showed the highest TFC value. The results for the other three fractions indicated TFC values in the order of CH_2_Cl_2_, *n*-BuOH fractions, and H_2_O residue.

#### 3.1.3. Antioxidant and Anti-Diabetic Activities of the Leaves of *Morus bombycis*

To evaluate antioxidant activity, the MeOH extract of three species and its organic solvent fractions from *M. bombycis* were tested via DPPH and ABTS radicals (Table 2). Among its organic solvent fractions, the EtOAc fraction showed the strongest scavenging activities against ABTS and DPPH. In vitro inhibitory activity assays by α-glucosidase and PTP1B were performed to evaluate the anti-diabetic effect of MeOH extract and four organic solvent fractions of *M. bombycis* leaves and isolated compounds. As given in Table 2, the MeOH extract and its four organic solvent fractions showed significant α-glucosidase inhibitory activities, compared to acarbose as a positive control. The EtOAc fraction, which showed significant α-glucosidase, exhibited good PTP1B inhibitory activity, compared to ursolic acid, although the CH_2_Cl_2_ fraction showed stronger inhibitory activity. According to the results of antioxidant and anti-diabetic activities, the EtOAc fraction was selected as a potent candidate, and further phytochemical isolation experiments were performed.

### 3.2. Evaluation of Bioactivities of Compounds Derived from the Leaves of Morus bombycis

#### 3.2.1. Antioxidant, Anti-Diabetic, and Anti-Diabetic Complication Activities of Compounds

As given in Table 3, tested compounds exhibited significant ONOO^−^ scavenging activity, with IC_50_ values ranging from 0.92 to 8.64 μM. In particular, compound **5** showed strong ONOO^−^ scavenging activity, compared to L-penicillamine as a positive control. Interestingly, tested compounds exhibited a significant α-glucosidase inhibitory effect, compared to acarbose as a positive control: Compound **4** showed the highest α-glucosidase inhibitory effect, followed by compounds **8**, **6**, **7**, **3**, and **5**. As for anti-diabetic activity by evaluation of the tested compounds on PTP1B inhibitory activities, compound **6** showed the highest inhibitory activity, followed by compounds **8**, **4**, and **7**. Compounds **4**, **6**, and **8** showed stronger inhibitory activity compared to ursolic acid, a positive control. In order to evaluate anti-diabetic complication activity, inhibitory activities of the tested compounds against BSA-AGEs formation and HRAR were determined. As given in Table 4, the test compounds except for compounds **7** and **8** demonstrated strong inhibitory activity, when compared to the positive control. In the case of HRAR inhibitory activity, compound **4** showed strong inhibitory activity, followed by compound **6**. These can be compared to quercetin as a positive control with an IC_50_ value of 16.67 μΜ. With regard to the above results, compounds **4**, **6**, **7**, and **8** might be promising candidates for anti-diabetic and anti-diabetic complication remedies, and further investigation was accomplished.

#### 3.2.2. Enzyme Kinetic Study of Isolated Compounds Derived from *Morus bombycis* Leaves

**Figure 3:**
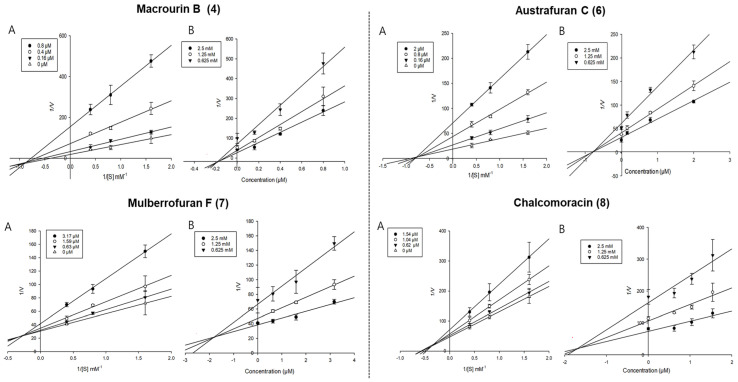
Lineweaver-Burk plots (**A**) and Dixon plots (**B**) of α-glucosidase inhibition by compounds **4** and **6**–**8**.

**Figure 4:**
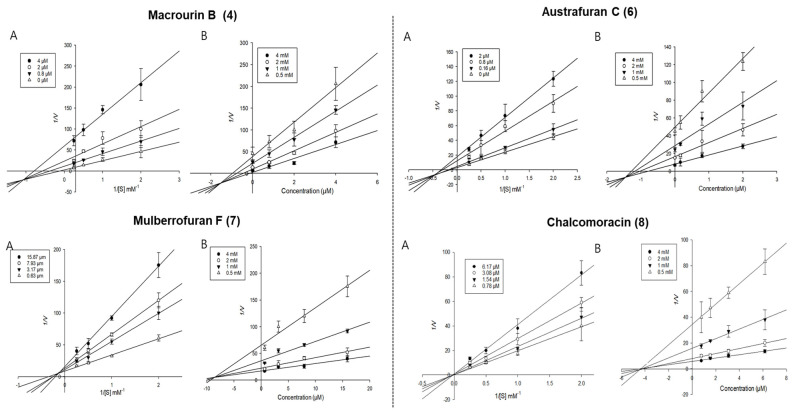
Lineweaver-Burk plots (**A**) and Dixon plots (**B**) of PTP1B inhibition by compounds **4** and **6**–**8**.

Enzyme kinetic analysis was performed with different concentrations of substrate (*p*NPG and *p*NPP) and various concentrations of compounds to determine the type of inhibition on the compounds. Lineweaver–Burk and Dixon plots were used to determine the type of inhibition in enzyme kinetics. Each line of inhibitors intersected at the *xy*-side, indicating mixed-type inhibitors. On the other hand, the lines penetrated the same point on the *x*-intercept, representing non-competitive inhibitors in Lineweaver–Burk plots, and the Dixon plot was also used to calculate the *K*_i_ value for the enzyme inhibitor complex with the value shown on the *x*-axis indicating the -*K*_i_ value [50,51,52]. Figure 3 depicts the enzyme kinetic analysis for α-glucosidase inhibition of each compound (**4** and **6**–**8**), with A representing the Lineweaver–Burk plot and B representing the Dixon plot. As displayed in Table 3 and Figure 3, compounds **4** and **6**–**8** exhibited mixed-type inhibition against α-glucosidase with respective *K*_i_ values of 0.19, 0.75, 1.71, and 1.84. In the enzyme kinetic analysis for PTP1B inhibition (Table 3 and Figure 4), compounds **4**, **6**, and **7** represented mixed-type inhibition with *K*_i_ values of 1.54, 1.45, and 8.90, respectively, while compound **8** exhibited non-competitive-type inhibition with *K*_i_ values of 4.41.

#### 3.2.3. Docking Interaction between Compounds and Key Binding Ligands of α-Glucosidase

**Figure 5:**
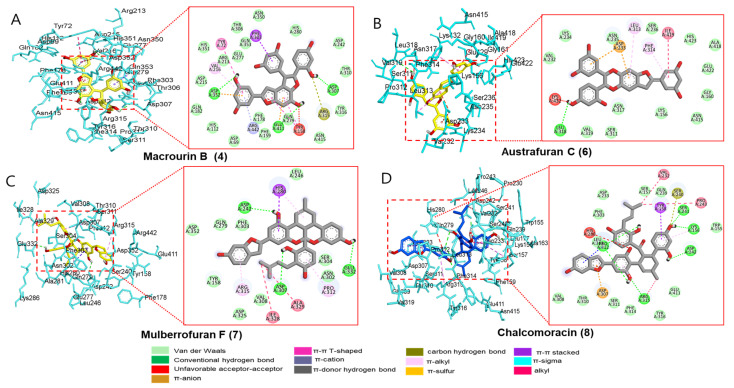
Molecular docking simulation models for α-glucosidase inhibition at the catalytic site by macrourin B (**A**), austrafuran C (**B**), mulberrofuran F (**C**), and chalcomoracin (**D**). 3D (**left**) and 2D (**right**) docking simulations were shown at each compound. Interaction was differentiated by various color (**bottom**).

Since compounds **4** and **6**–**8** exhibited significant inhibitory activities against α-glucosidase and PTP1B (which play important enzymes in therapeutic strategy against DM), four candidates were subjected to a molecular docking analysis. All the docked active compounds overlapped within the α-glucosidase (PDB: 3A4A) pocket sites, and α-D-glucose was used as a co-crystalline ligand for α-glucosidase. Using AutoDock Vina, the ligand–enzyme complexes of the four test compounds, acarbose and α-D-glucose, were stably posed in the catalytic pocket of α-glucosidase (Figure 5A−D). Hydrogen bonds, hydrophobic interactions, and electrostatic interactions were used to calculate the binding energies of test compounds. The predicted binding energies and binding residues are provided in Table 5. As given in Table 5 and Figure 5A, the binding energy of α-glucosidase enzyme−macrourin B (**4**) complex was −11.1 kcal/mol, indicating the presence of three hydrogen bonds with interacting residues such as Asp352, Asp307, and Glu411 within the active pocket of α-glucosidase. Other important amino acid residues, Arg315, Val216, Tyr158, Phe303, Tyr158, and Tyr72, also interacted with compound **4**. In addition to electrostatic interaction, macrourin B (**4**) interacted with the Arg442 and Asp352 residues. The α-glucosidase enzyme−austrafuran C (**6**) complex showed a binding energy of −9.3 kcal/mol, indicating the presence of a conventional hydrogen bond with Leu318 within the pocket site of α-glucosidase, while various key amino acid residues, including Leu313 and Phe314, exhibited π-alkyl interactions. Lys432, Ile419, Phe314, and Leu313 residues showed hydrophobic interactions, and the Asp233 residue exhibited electrostatic interactions with compound **6** (Table 5 and Figure 5B). The binding energy of α-glucosidase enzyme−chalcomoracin (**7**) complex was -10.6 kcal/mol, indicating the presence of three hydrogen bonds with Asp242, Glu332, and Asp307, as well as hydrophobic bonding with Ala329, Ile328, Arg315, Pro312, and His280 within the active pocket site of α-glucosidase (Table 5 and Figure 5C). As given in Table 5 and Figure 5D, the α-glucosidase enzyme−mulberrofuran F (**8**) complex had the lowest binding energy of −11.5 kcal/mol due to (i) the presence of five hydrogen bonds with the interacting residues (Arg315, Asp242, Lys156, Ser241, and Pro312), (ii) hydrophobic bonding with His280, Ser240, Tyr158, Pro243, Val232, Arg315, and Pro312, and (iii) electrostatic interactions with Asp307.

The positive controls (acarbose) used in the bioassay were also docked within α-glucosidase active sites and revealed hydrogen bonding with Asp352, Asp215, Arg442, Gln279, Pro312, Ser240, and Tyr158 with varying bond lengths (data are not shown). In addition to hydrogen bonds, acarbose revealed carbon–hydrogen bonds, alkyl, π-sigma, and unfavorable acceptor–acceptor interactions with Pro312, His280, and Glu411 within the active pocket of 3A4A. Next, the probable binding mode of co-crystalline ligand of 3A4A (α-D-glucose) exhibited several important interactions, such as conventional hydrogen bonds with Asp69, Arg442, Arg213, Asp352, Asp215, Glu277, His112, and His351 within the active pocket of α-glucosidase. Other key residues (Tyr72 and Asp69) revealed π-donor–hydrogen bond and carbon–hydrogen bond interactions, respectively. The putative binding and important interactions of all compounds are shown in Figure 5, where 3D interactions revealed better insight into these compounds within the active pocket of α-glucosidase.

#### 3.2.4. Docking Interaction between Compounds and Key Binding Ligands of PTP1B

**Figure 6:**
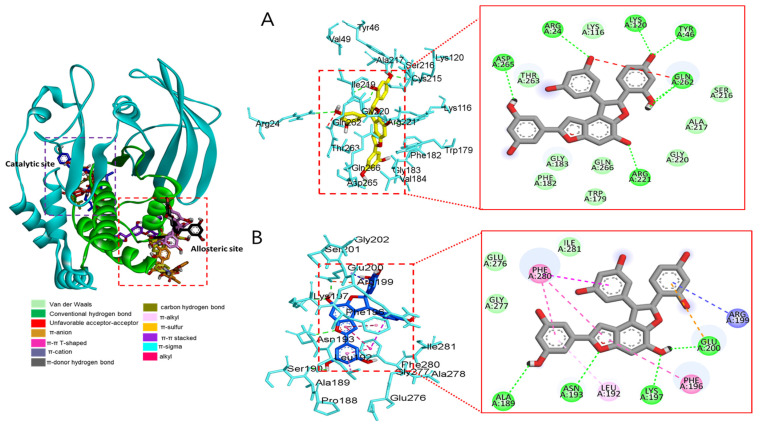
Molecular docking simulation models for PTP1B inhibition by macrourin B at catalytic site (**A**) and allosteric site (**B**). 3D (**left**) and 2D (**right**) docking simulations were shown at each binding site. Interaction was differentiated by various color (**left bottom**).

**Figure 7:**
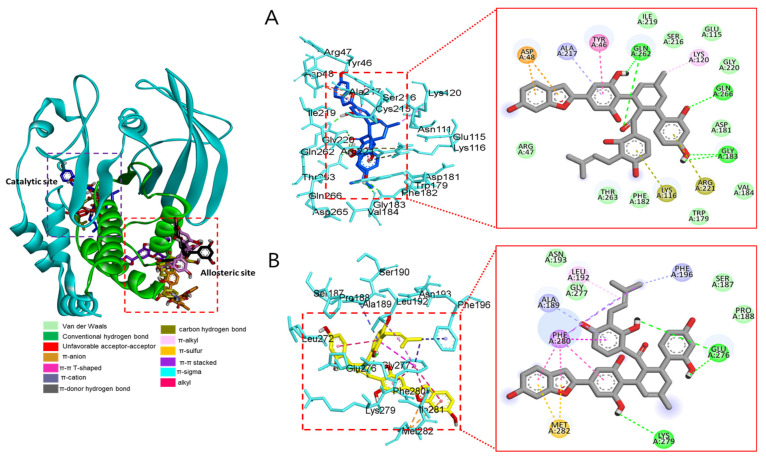
Molecular docking simulation models for PTP1B inhibition by chalcomoracin at catalytic site (**A**) and allosteric site (**B**). 3D (**left**) and 2D (**right**) docking simulations were shown at each binding site. Interaction was differentiated by various color (**left bottom**).

**Figure 8:**
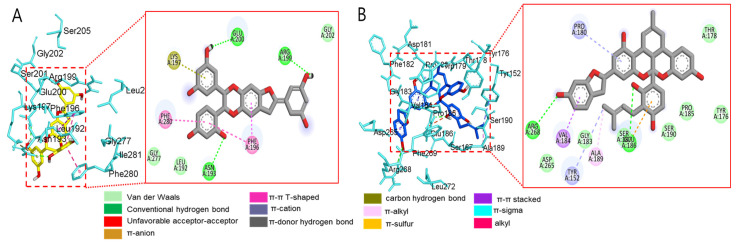
Molecular docking simulation models for PTP1B inhibition at the allosteric site by austrafuran C (**A**) and mulberrofuran F (**B**). 3D (**left**) and 2D (**right**) docking simulations were shown at each compound. Interaction was differentiated by various color (**left bottom**).

Molecular docking analysis of compounds **4** and **6**–**8** against PTP1B (PDB: 1T49) was also performed using AutoDock Vina. Compound 2 and compound 23 were used as co-crystalline ligands for PTP1B, and ligands were extracted from their crystal structures of proteins. The active sites of in silico PTP1B contain the common structural motif of protein tyrosine phosphatase (PTP), and the catalytic sites are His-Cys-Ser-Ala-Gly-Iso-Gly-Arg, which have major structural features [57]. The ligand–enzyme complexes of the four test compounds were suitably posed in the allosteric and catalytic sites of PTP1B (Figure 6, Figure 7 and Figure 8), and the results of molecular docking scores are summarized in Table 6. In catalytic inhibition mode, the PTP1B enzyme−macrourin B (**4**) complex exhibited significant negative binding energy of −8.7 kcal/mol due to six hydrogen bonds with the interacting residues, Arg24, Arg221, Asp265, Gln262, Lys120, and Tyr46, as well as hydrophobic bonding with Gln262 within the catalytic pocket (Figure 6A). Allosteric inhibition of compound **4** against PTP1B also exhibited the lowest binding energy of −9.0 kcal/mol. This was due to (i) the presence of four hydrogen bonds with interacting residues (i.e., Ala189, Asn193, Glu200, and Lys197), (ii) hydrophobic bonding with Leu192, Arg199, Phe280, and Phe196, and (iii) electrostatic interactions with Glu200 (Figure 6B). As shown in Figure 7A, the catalytic inhibition mode of the PTP1B enzyme−chalcomoracin (**8**) complex exhibited the lowest binding energy with -8.9 kcal/mol, indicating the presence of three hydrogen bonds with the interacting residues (Gly183, Gln266, and Gln262), hydrophobic bonding with Ala217, Lys120, and Tyr46, and electrostatic interactions with Lys116, Arg221, and Asp48 in the catalytic pocket. In allosteric inhibition mode (Figure 7B), the PTP1B enzyme−chalcomoracin (**8**) complex exhibited a binding energy of −8.4 kcal/mol, indicating the presence of two hydrogen bonds with Glu276 and Lys279 as well as hydrophobic inhibition with Leu192, Ala189, Phe196, Phe280, and Met282. As shown in Figure 8A, the allosteric binding energy of the PTP1B enzyme−austrafuran C (**6**) complex was −7.9 kcal/mol due to (i) multiple conventional hydrogen bonds with Asn193, Glu200, and Arg199, (ii) hydrophobic bonding with Phe196 and Phe280, and (iii) electrostatic interactions with Lys197 (π-cation) within the allosteric pocket (Figure 8A). The PTP1B enzyme−mulberrofuran F (**7**) complex in allosteric mode exhibited a binding energy of −8.1 kcal/mol, indicating the presence of two hydrogen bonds with Arg268 and Glu186, hydrophobic bonding with Pro180, Tyr152, Ala189, and Val184, and electrostatic interactions with Glu186 in the allosteric pocket (Figure 8B).

Moreover, the probable binding mode of co-crystalline ligand compound 2 (yellow) as an allosteric inhibitor exhibited several important interactions, including hydrogen bonding with Asn193 and Glu276 within the allosteric site of PTP1B (combined docking model is be on the left at Figure 6 and Figure 7). Other important residues, such as Lys197, and Leu192 revealed alkyl interactions, and Ala189, Leu192, Phe280, and Phe196 showed π-alkyl interactions. Halogen interactions with Glu200, π-π T-shaped interactions with Phe280, π-sigma interactions with Phe196, and π-π stacked interactions with Phe280 were noticed when compound 2 was docked inside PTP1B. The plausible binding mode of co-crystalline ligand compound 23 as a catalytic inhibitor exhibited numerous key interactions, such as conventional hydrogen bonding with Asp48, Arg254, Arg221, Ser216, Gly220, Gly218, Ile219, and Ala217 within the catalytic site of PTP1B (data are not shown). In addition, π-alkyl interactions were observed between compound 23 and Ala27 and Ala217. Some amino acids exhibited π-sigma interactions like Tyr46 and Ala217. Others showed π-sulfur interactions with Met258, π-π stacked interactions with Tyr46, carbon–hydrogen bonds with Tyr46, and π-donor–hydrogen bond interactions with Gly220. The binding site and interactions of all the compounds are provided in Table 6 and Figure 6, Figure 7 and Figure 8. Similar to the in vitro enzyme kinetic inhibition, compounds **4** and **6**–**8** exhibited good docking results and binding energies (Table 5 and Table 6), which help explain the in vitro results.

## 4. Discussion

About 95% of people with DM have T2DM, which is caused by the inefficient use of insulin in the body. Insufficient insulin production and insulin resistance are the causes of T2DM, and these affect the control of the metabolism of proteins, lipids, and carbohydrates. Deterioration of insulin-producing pancreatic ß-cells and insulin resistance present in diverse organs contribute to microvascular and macrovascular problems [1,2]. Consequently, chronic and accelerated hyperglycemia cause cardiovascular disease, coronaropathy, and other problems, especially diabetic retinopathy, and diabetic foot [9]. Moreover, free radicals and ROS are produced by living things as part of regular physiological and biochemical processes, and their overproduction can lead to oxidative damage to biomolecules (such as lipids, proteins, and DNA) and many chronic diseases in people, including DM, Alzheimer’s disease, cardiovascular disease, and chronic inflammation [16]. Since the enzymes PTP1B, α-glucosidase, and AR (as well as non-enzymatic glycation products known as AGEs) play critical roles in T2DM, much research has been conducted to develop therapeutic inhibitors. Unfortunately, clinical trials using enzyme inhibitors, which are important targets of T2DM mechanisms, have recently failed to produce effective therapy agents [58]. For example, aminoguanidine has been used to inhibit DM complications, it has adverse effects on the heart and lungs, and may cause histamine buildup in the system [59]. Therefore, therapeutic agents isolated from natural products that are utilized in conventional medicine or functional foods may be effective treatments for DM.

Several active compounds and the EtOAc fraction derived from *M. bombycis* leaves against DM were found, and comparisons were made on the antioxidant and anti-diabetic effects among three *Morus* species (e.g., *M. alba* and *M. lhou*). The antioxidant and anti-diabetic effects of *M. bombycis* extract showed significantly higher inhibitory activity than those of the two *Morus* species, such as *M. alba* and *M. lhou* (Table 1). In the phytochemical content evaluation of the leaves of *M. bombycis*, the EtOAc fraction indicated the highest value in TPC and TFC; potent inhibitory activities against both α-glucosidase and PTP1B (Table 2). Repeated column chromatography of potent bioactive EtOAc fractions led to the isolation of compounds **1**–**8**, and we further evaluated their bioactivities. Overall, the goal of this study was to quantitatively analyze the EtOAc fraction from *M. bombycis* leaves and evaluate its antioxidant, anti-diabetic, and anti-diabetic complication effects.

Compounds **1** and **2** have antioxidant [60], anti-diabetic [61], and anti-inflammatory effects [62]. Kwon et al. [49] revealed that compounds **3** and **5** have a strong inhibitory effect on diabetics and diabetic complications and can scavenge free radicals. Compound **7** has potent scavenging activity against Fe^2+^/cysteine-induced microsomal lipid peroxidation assay [63]. Compound **8** has been studied for the anti-diabetic activity to inhibit α-glucosidase [31] and PTP1B [64].

Although there have been many studies on compounds isolated from *M. bombycis* leaves, compound **6** has not been investigated by bioactivity screening, and little research has been conducted on a 2-arylbenzofuran-type compound (**4**), a stilbene-dimer-type compound (**6**), and the Diels–Alder type adducts (**7**, **8**), which are known to have antioxidant and anti-diabetic properties. Compounds **4**–**6** and **8** exhibited potent scavenging activities against ONOO^−^. All test compounds demonstrated significant α-glucosidase inhibitory activity when compared to a positive control, acarbose. Compounds **4** and **6**–**8** were strong PTP1B inhibitors by compared to a positive control, ursolic acid. The inhibitory activities of AGE formation were tested to confirm the anti-diabetic complications effect. All the compounds except for compound **7** demonstrated significant inhibitory effects, and compounds **3** and **4** exhibited extremely potent inhibitory activities against the formation of AGEs compared to the positive control. Moreover, compounds **4** and **6** exhibited significant inhibitory activities against HRAR. Compounds **3** and **5** were recently reported to have anti-diabetic and antioxidant activities by inhibiting α-glucosidase, PTP1B, AGEs, and ONOO^−^ [49], which is consistent with our current studies. 

Since compounds **4** and **6**–**8** have the potential to be effective α-glucosidase and PTP1B inhibitors, we focused on the anti-DM effects of four key compounds by performing enzyme kinetic studies and molecular docking simulations. Studying the impact of the inhibitory concentration on enzyme kinetics is crucial to comprehending the mechanism of inhibitor-mediated enzyme inhibition. The inhibitors have an affinity for the enzyme binding site in which greater affinities are indicated by lower values of *K*_i_. All the test compounds used in the enzyme kinetic study against α-glucosidase were mixed-type inhibitors. Mixed-type compounds may be posed at active and/or allosteric sites, while non-competitive compound **8** could be posed at the allosteric site on PTP1B.

Molecular docking is a way to determine how a ligand will fit into a protein’s binding site. A scoring function is used to determine the binding energy values for each structure to predict the activity of the bound ligand [65]. In silico docking simulation studies on test compounds were performed to demonstrate the α-glucosidase and PTP1B inhibition mechanisms of the potent bioactive compounds (Figure 5, Figure 6, Figure 7 and Figure 8). The docking scores of the binding energies on each enzyme were estimated and are listed in Table 5 and Table 6. The results of the docking simulation against α-glucosidase confirmed that all test compounds had high affinity and lower binding energies within the enzyme catalytic site compared to acarbose (Figure 5 and Table 5). Previous research has supported the existence of catalytic residues on α-glucosidase, such as Asp215, Glu277, Asp352, His112, Asp242, Gln279, and His280, which play an important role in inhibiting enzyme activation [26]. Among them, the Diels–Alder type adducts (**7** and **8**) and two stilbene dimers (**4** and **6**) interacted via hydrogen bonds to α-glucosidase catalytic residues. Furthermore, the 12-OH and 6-OH of compound **4** interacted with the Asp442 residue and the hydrogen bonding with Asp352 and Glu411 residues on the α-glucosidase catalytic site. Compound **8** interacted with Tyr158 residue via the hydrophobic interaction as well as Pro312 residue via the hydrogen bonds. None of the test compounds were bound to the residue to which the substrate, α-D-glucose, binds via hydrogen bonding. Compounds **4** and **8** interacted with binding residues similar to acarbose in terms of hydrogen bond interactions. In other words, the findings of this study showed that compounds **4** and **8** interact with major residues without causing adverse effects compared to acarbose (which is known to have side effects). Compounds **4** and **8** had the lowest energy and inhibited the α-glucosidase when the docking score of residues that interacted with the target was calculated in this manner.

To demonstrate the interaction and binding modes of active compounds with PTP1B, these test compounds were compared with the reported compounds **2** (allosteric inhibitor) and **23** (catalytic inhibitor). The calculated docking score of binding energies indicated high affinity and lower binding energies within the enzyme catalytic site and allosteric site. All test compounds posed within the allosteric site on PTP1B interacted with multiple hydrogen bonds for compounds **4** and **6**–**8** (Figure 6, Figure 7 and Figure 8). Similar to previous α-glucosidase studies, compound **4** was combined with the same residue as allosteric and catalytic inhibitors to inhibit PTP1B (Table 6). In particular, stilbene dimer **4** and Diels–Alder type adduct **8** interacted with hydrogen bonds. As shown in Figure 7 and Figure 8, compounds **6** and **8** showed interactions by binding to Asn 193 and Glu 276—the same residues as allosteric inhibitors. Previous research by Jung et al. [66] found that Cys215, His214, Arg221, Thr177, Pro189, Glu186, Glu200, Ser201, Gly209, Ala264, and Ile281 residues play a role in the catalytic loop of PTP1B, which is consistent with our findings.

We have demonstrated that compounds **4** and **6**–**8**, which were isolated at first from *M. bombycis* leaves, have anti-diabetic and anti-diabetic complication effects. Kinetic analyses and a molecular docking study were used to determine the interaction mechanisms within the enzyme sites. The implication of these findings is noteworthy in that the inhibition mechanism of stilbene dimers (**4** and **6**) and Diels–Alder adducts (**7** and **8**) against DM and its complications corroborate their potential as therapeutic or preventive agents and functional foods.

## 5. Conclusions

*Morus bombycis* has been used as traditional medicine, and many studies have been conducted on other parts of the plant such as the root, bark, and cortex. However, few studies have explored *M. bombycis* leaves. In the present study, we found that *M. bombycis* and its isolated compounds were very effective scavengers/inhibitors against ONOO^−^, α-glucosidase, PTP1B, AGEs, and HRAR. Among the test compounds, stilbene-dimer-type compounds **4** and **6** exhibited strong antioxidant, anti-diabetic, and anti-diabetic complication effects, whereas Diels–Alder type adducts compounds **7** and **8** effectively inhibited α-glucosidase and PTP1B. All the tested compounds showed mixed-type inhibition against α-glucosidase in the enzyme kinetic study. Compounds **4** and **6**–**7** were confirmed as mixed-type inhibitors, while compound **8** was determined to be a non-competitive inhibitor against PTP1B. Compounds **4** and **6**–**8** docked within the catalytic site of α-glucosidase, whereas compounds **4** and **8** were bound within both the catalytic and allosteric sites of PTP1B, and compounds **6** and **7** were bound only within the allosteric site. In conclusion, these findings imply that stilbene dimers and Diels–Alder type adducts could be novel and/or important natural inhibitors or preventive resources as antioxidants, anti-diabetic agents, and anti-diabetic complication agents.

## Figures and Tables

**Table 1:** Comprehensive comparison of the MeOH extracts of the leaves of three dominant Morus species on TPC/TFC analysis and DPPH/ABTS radical scavenging and α-glucosidase inhibitory activities.

Species	TPC (mg GAE/g) ^a^	TFC (mg CE/g) ^a^	ABTS IC_50_ (μg/mL) ^a^	DPPH IC_50_ (μg/mL) ^a^	α-Glucosidase IC_50_ (μg/mL) ^a^
*Morus alba*	47.91 ± 0.08	74.80 ± 0.47	72.25 ± 0.75	49.83 ± 2.87	319.26 ± 22.71
*Morus lhou*	57.82 ± 0.52	112.93 ± 0.88	132.49 ± 8.24	24.25 ± 0.59	148.24 ± 16.29
*Morus bombycis*	62.67 ± 0.18	87.70 ± 1.67	95.74 ± 19.52	24.30 ± 0.62	26.35 ± 2.98
Trolox ^b^			2.72 ± 0.13		
L-ascorbic acid ^b^			3.38 ± 0.19	2.82 ± 0.57	
Acarbose ^b^					352.09 ± 22.27

^a^ The values are expressed as the mean ± SD of triplicate experiments. ^b^ Positive controls were used in each assay.

**Table 2:** TPC/TFC values and ABTS/DPPH radical scavenging activities and α-glucosidase/PTP1B enzyme inhibitory activities of extract and its organic solvent fractions from *Morus bombycis* leaves.

Species	TPC (mg GAE/g) ^a^	TFC (mg CE/g) ^a^	ABTS IC_50_ (μg/mL) ^a^	DPPH IC_50_ (μg/mL) ^a^	α-Glucosidase IC_50_ (μg/mL) ^a^	PTP1B IC_50_ (μg/mL) ^a^
MeOH ext.	62.67 ± 0.18	87.70 ± 1.67	95.74 ± 19.52	24.30 ± 0.62	26.35 ± 2.98	24.71 ± 2.92
CH_2_Cl_2_ fr.	55.71 ± 1.17	208.45 ± 0.98	85.16 ± 13.13	115.34 ± 25.41	57.67 ± 2.01	7.09 ± 0.72
EtOAc fr.	261.59 ± 3.58	260.43 ± 9.07	12.84 ± 1.23	4.66 ± 0.65	6.74 ± 1.57	25.17 ± 1.66
*n*-BuOH fr.	138.18 ± 0.72	173.16 ± 0.93	18.75 ± 0.94	7.60 ± 0.36	18.63 ± 1.60	46.32 ± 3.39
H_2_O fr.	40.45 ± 1.82	37.96 ± 1.30	98.65 ± 11.42	37.12 ± 1.18	39.73 ± 9.04	115.52 ± 12.44
Trolox ^b^			2.72 ± 0.13			
L-ascorbic acid ^b^			3.38 ± 0.19	2.82 ± 0.57		
Acarbose ^b^					352.09 ± 22.27	
Ursolic acid ^b^						6.39 ± 0.42

^a^ The values are expressed as the mean ± SD of triplicate experiments. ^b^ Positive controls were used in each assay.

**Table 3:** Inhibitory activities of isolated compounds from *Morus bombycis* leaves against α-glucosidase and PTP1B.

Test Compounds	Peroxynitirite	α-Glucosidase			PTP1B		
	IC_50_ (μM) ^a^	IC_50_ (μM) ^a^	Inhibition Mode ^c^	Inhibition Constant (*K*_i_) ^d^	IC_50_ (μM) ^a^	Inhibition Mode ^c^	Inhibition Constant (*K*_i_) ^d^
Oxyresveratrol (**3**)	6.24 ± 0.06	2.58 ± 0.23	-		72.88 ± 1.87	-	
Macrourin B (**4**)	2.61 ± 0.13	0.44 ± 0.03	Mixed	0.19	2.50 ± 0.17	Mixed	1.54
Moracin M (**5**)	0.92 ± 0.13	6.11 ± 0.53	-		27.14 ± 4.20	-	
Austrafuran C (**6**)	2.76 ± 0.25	1.01 ± 0.17	Mixed	0.75	1.69 ± 0.02	Mixed	1.45
Mulberrofuran F (**7**)	8.63 ± 0.02	1.22 ± 0.05	Mixed	1.84	10.53 ± 0.25	Mixed	8.90
Chalcomoracin (**8**)	3.03 ± 0.53	0.98 ± 0.03	Mixed	1.71	2.06 ± 0.39	Non-competitive	4.41
L-Penicillamine ^b^	0.62 ± 0.17						
Acarbose ^b^		321.46 ± 21.13					
Ursolic acid ^b^					13.53 ± 0.18		

^a^ The values are expressed as the mean ± SD of triplicate experiments. ^b^ Positive controls were used in each assay. ^c^ Inhibition types were determined by interpretation of Lineweaver–Burk plots. ^d^ Inhibition constants (*K*_i_) were determined by interpretation of the Dixon plots.

**Table 4:** Inhibitory activities of AGEs formation and HRAR of isolated compounds of EtOAc fraction from *Morus bombycis* leaves.

Test Compounds	BSA-AGEs IC_50_ (μM) ^a^	HAS-AGEs IC_50_ (μM) ^a^	HRAR IC_50_ (μM) ^c^
Oxyresveratrol (**3**)	10.36 ± 0.39	5.38 ± 0.26	264.8
Macrourin B (**4**)	9.44 ± 0.18	7.98 ± 0.62	<4
Moracin M (**5**)	2.40 ± 0.18	2.07 ± 0.03	238.4
Austrafuran C (**6**)	13.74 ± 0.55	6.15 ± 0.25	26.78
Mulberrofuran F (**7**)	ND	ND	337.3
Chalcomoracin (**8**)	137.60 ± 0.33	112.59 ± 3.62	265.0
Aminoguanidine ^b^	581.03 ± 28.67	504.07 ± 14.92	
Quercetin ^bc^			16.67

^a^ The values are expressed as the mean ± SD of triplicate experiments. ^b^ Positive controls were used in each assay. ^c^ HRARs are expressed as one result of a single experiment. ND, not detected at tested concentration.

**Table 5:** Binding site residues and docking scores of compounds in the α-glucosidase using the AutoDock Vina 4.2 program.

Compounds	Number of H-Bonds	Binding Energy (kcal/mol)	Hydrogen Bonds Interacting Residues	Hydrophobic Interacting Residues	Electrostatic Interacting Residues
Macrourin B (**4**)	3	−11.1	Asp352, Asp307, Glu411	Arg315 (carbon–hydrogen bond), Val216 (π-alkyl), Tyr158 (unfavorable acceptor–acceptor), Phe303 (π-π stacked), Tyr158 (π-π T-shaped), Tyr72 (π-π T-shaped)	Arg442 (π-cation), Asp352 (π-anion)
Austrafuran C (**6**)	1	−9.3	Leu318	Lys432 (unfavorable donor–donor), Ile419 (alky), Phe314 (π-alkyl), Leu313 (π-alkyl)	Asp233 (π-anion)
Mulberrofuran F (**7**)	3	−10.6	Asp242, Glu332, Asp307	Ala329 (alkyl), Ile328 (alkyl), Arg315 (π-alkyl), Pro312 (π-alkyl), His280 (π-alkyl), His280 (π-π stacked)	
Chalcomoracin (**8**)	5	−11.5	Arg315, Asp242, Lys156, Ser241, Pro312	His280 (unfavorable acceptor–acceptor), Ser240 (carbon–hydrogen bond), Tyr158 (π-π stacked), Pro243 (alkyl), Val232 (alkyl), Arg315 (alkyl), Arg315 (π-alkyl), Lys156 (π-alkyl), Pro312 (π-sigma)	Asp307 (π-anion),
Acarbose	7	−8.2	Asp352, Asp215, Arg442, Gln279, Pro312, Ser240, Tyr158	Pro312 (carbon–hydrogen bond), His280 (π-sigma), Glu411 (unfavorable acceptor–acceptor)	
Alpha-D-glucose	8	−6.8	Asp69, Arg442, Arg213, Asp352, Asp215, Glu277, His112, His351	Tyr72 (π-donor–hydrogen bond), Asp69 (carbon–hydrogen bond)	

**Table 6:** Binding site residues and docking scores of compounds in the PTP1B using the AutoDock Vina 4.2 program.

Compounds	Number of H-Bonds	Binding Energy (kcal/mol)	Hydrogen Bonds Interacting Residues	Hydrophobic Interacting Residues	Electrostatic Interacting Residues
Macrourin B (**4**)	6	−8.7	Arg24, Arg221, Asp265, Gln262, Lys120, Tyr46	Gln262 (unfavorable acceptor–acceptor)	
Austrafuran C (**6**)	4	−9.0	Ala189, Asn193, Glu200, Lys197	Leu192 (π-alkyl), Arg199 (amide-π stacked), Phe280 (π-π T-shaped), Phe280 (π-π stacked), Phe196 (π-π stacked)	Glu200 (π-anion)
Mulberrofuran F (**7**)	3	−7.9	Asn193, Glu200, Arg199	Phe196 (π-π T-shaped), Phe196 (π-π stacked), Phe280 (π-π stacked)	Lys197 (π-cation),
Chalcomoracin (**8**)	2	−8.1	Arg268, Glu186	Pro180 (π-alkyl), Tyr152 (π-alkyl), Ala189 (alkyl), Val184 (π-sigma),	Glu186 (π-anion),
Compound 2 (allosteric inhibitor)	3	−8.9	Gly183, Gln266, Gln262	Ala217 (π-alkyl), Lys120 (alkyl), Tyr46 (π-π stacked)	Lys116 (π-cation), Arg221 (π-cation), Asp48 (π-anion)
Compound 23 (catalytic inhibitor)	2	−8.4	Glu276, Lys279	Leu192 (alkyl), Ala189 (π-alkyl), Phe196 (π-alkyl), Phe280 (π-alkyl), Met282 (π-sulfur), Phe280 (π-sigma), Phe280 (π-π T-shaped), Phe280 (π-π stacked)	

## Data Availability

Data is contained within the article and Supplementary Material.

## Supplementary Materials

The following supporting information can be downloaded at https://www.mdpi.com/article/10.3390/antiox12040837/s1, Table S1: Inhibitory activities of subfractions of EtOAc fraction from *Morus bombycis* leaves against peroxynitrite, α-glucosidase, PTP1B and AGE formation; Table S2: NMR spectra of isolated compounds **4** and **6**. Table S3. The 1D NMR data of compounds **7** and **8**. Figure S1. The NMR chromatograms of compound **4**. Figure S2. The NMR chromatograms of compounds **6**, **7**, and **8**. Table S4. The 1D NMR data of compounds **1**–**3**.
