# Supplementary material for: An Arylbenzofuran, Stilbene Dimers, and Prenylated Diels–Alder Adducts as Potent Diabetic Inhibitors from Morus bombycis Leaves
Source: Antioxidants (Basel). 2023 Mar 30;12(4):837. doi: 10.3390/antiox12040837 (PMC10134988; doi:10.3390/antiox12040837)
Supplement: Supplementary file 1 [file antioxidants-12-00837-s001.zip › antioxidants-2304993-supplementary.pdf]

Supplementary Materials

An Arylbenzofuan, Stilbene Dimers and Prenylated Diels-Alder Adducts as Potent Diabetic Inhibitors from *Morus bombycis* Leaves

Seon Min Ju[1], Seung Mi Ko[1], Jung Hye Ryu[1], Md Yousof Ali[2,3], Jae Sue Choi[2, *], Hyun Ah Jung[1, *]

[1]Department of Food Science and Human Nutrition, Jeonbuk National University, Korea; [2]Department of Food Science and Nutrition, Pukyong National University, Korea; [3]Department of Physiology and Pharmacology, Hotchkiss Brain Institute and Alberta Children's Hospital Research Institute, Cumming School of Medicine, University of Calgary, Calgary, AB T2N 4N1, Canada

Correspondence: choijs@pknu.ac.kr (J.S.C.); jungha@jbnu.ac.kr (H.A.J.); Tel.: +82-51-629-5845 (J.S.C.); +82-63-270-4882 (H.A.J.)

Table S1. Inhibitory activities of subfractions of EtOAc fraction from *Morus bombycis* leaves against peroxynitrite, α-glucosidase, PTP1B and AGE formation

Subfractions	Peroxynitirite IC_{50} (μg/mL)[a]	α-Glucosidase IC_{50} (μg/mL)[a]	PTP1B IC_{50} (μg/mL)[a]	BSA-AGEs IC_{50} (μg/mL)[a]
E2	1.23 ± 0.01	249.58 ± 18.53	56.93 ± 4.46	6.12 ± 0.29
E3	< 0.032	10.22 ± 2.29	25.11 ± 4.46	14.65 ± 1.50
E4	0.13 ± 0.02	5.11 ± 1.87	14.28 ± 5.46	4.19 ± 0.37
E6	0.34 ± 0.01	1.30 ± 0.08	4.65 ± 0.56	3.58 ± 0.24
E8	1.04 ± 0.09	0.77 ± 0.03	4.86 ± 1.40	4.60 ± 0.05
L-Penicillamine[b]	0.67 ± 0.01		5.60 ± 0.77	
Acarbose [b]		224.27 ± 4.82		
Ursolic acid [b]			6.39 ± 0.42	
Aminoguanidine[b]				36.73 ± 1.64

[a]The values are expressed as the mean ± SD of triplicate experiments. [b]Positive control was used in each assay

Table S2. The 1D NMR data of compounds 4 and 6

	Macrourin B (**4**)			Austrafuran C (**6**)	
Position	H	C	Position	H	C
2		156.5	2		157.3
3	6.43 (br s, *J*=0.15Hz)	100.3	3	6.90 (1H, s)	102.1
3a		119.4	3a		124.1
4		121.6	4	7.07 (1H, s)	107.8
5		133.8	5		142.9
6		141.1	6		143.9
7	6.90 (s)	98.5	7	7.09 (1H, s)	99.8
7a		152.5	7a		151.2
8		146.2	1'		133.7
9	6.65 (d, *J*=2Hz)	103.8	2'	6.77 (d, J=2.5 Hz)	104
10		159.8	3'		160
11	6.19 (t, *J*=3.2Hz)	103.4	4'	6.25 (t, J=2.3 Hz)	103.7
12		159.8	5'		160
13	6.65 (d, *J*=2Hz)	103.8	6'	6.78 (d, *J*=2.5 Hz)	104
1'		119.5	1"		116
2'		157.5	2"		157.7
3'	6.33 (d, *J*=2.5Hz)	103.6	3"	6.18 (d, *J*=2.3 Hz)	103.4
4'		159.4	4"		159.7
5'	6.27 (dd, *J*=8.4Hz, 2.3Hz)	107.5	5"	6.27 (dd, *J*=8.4 Hz, 2.3 Hz)	108
6'	7.10 (d, *J*=8.5Hz)	129.3	6"	7.04 (d, *J*=8.5 Hz)	131
7'	5.78 (d, *J*=7.5Hz)	90.9	7"	5.23 (d, *J*=8Hz)	76.3
8'	4.74 (d, *J*=7Hz)	57	8"	5.07 (d, *J*=7.6Hz)	81
9'		145.9	9"		140.5
10'	6.23 (d, *J*=2.5Hz)	107.7	10"	6.22 (d, *J*=2.3Hz)	107.5
11'		159.5	11"		159
12'	6.17 (t, *J*=4.6Hz)	102.2	12"	6.13 (t, *J*=2.3 Hz)	103.4
13'		159.5	13"		159
14'	6.23 (d, *J*=2.5Hz)	107.7	14"	6.22 (d, *J*=2.3Hz)	107.5

Table S3. The 1D NMR data of compounds 7 and 8

Position	Mulberrofuran F (**7**)		Position	Chalcomoracin (**8**)	
	H	C		H	C
2		155.4	2		156.4
3	6.91 (d, *J*=1Hz)	102.3	3	6.91 (d, *J*= 2Hz, overlapping)	101.8
3a		123.1	3a		122.6
4	7.32 (d, *J*=8.5Hz)	122	4	7.34 (d, *J*=8.5Hz)	121.8
5	6.71 (dd, *J*=8Hz)	113.7	5	6.76 (m, overlapping)	113.1
6		156.9	6		157.8
7	6.88 (d, *J*=1.7Hz)	98.5	7	6.91 (d, *J*= 2Hz, overlapping)	98.3
7a		156.9	7a		155.4
1'		131.5	1'		130.9
2'	6.87 (d, *J*=3.4Hz)	106	2'	6.76 (m, overlapping)	104.8
3'		154	3'		156.5
4'		113.3	4'		115.9
5'		158.3	5'		156.5
6'	(m, solvent overlapping)	105.2	6'	6.76 (m, overlapping)	104.9
1"		134.1	1"		133.8
2"	6.41 (br d, *J*=5.5Hz)	123	2"	5.77 (br s)	124.3
3"	(m, solvent overlapping)	35.9	3"	4.11 (br s)	33.1
4"	(m, solvent overlapping)	38	4"	4.63 (t, *J*=4.20 Hz)	47.8
5"	(m, solvent overlapping)	29.1	5"	3.75 (t, *J*=5.7 Hz)	36.5
6"	2.69 (dd, *J*=15Hz)	36.5	6"	2.18 (d, *J*=18.5Hz)	32.2
6b"	2.06 (dd, *J*=15Hz)		6b"	2.50 (d, *J*=18Hz)	32.2
7"	1.73 (s)	23.9	7"	1.93 (br s)	23.8
8"		104.2	8"		209.8
9"		ND	9"		113.4
10"		155.6	10"		164.6
11"		117.8	11"		116.6
12"		157.3	12"		164.2
13"	6.23 (d, *J*=8.5Hz)	107.4	13"	6.43 (d, *J*=9Hz)	108.1
14"	7.11 (d, *J*=8.5Hz)	128	14"	8.43 (d, *J*=8.5Hz)	132.1
15"		117.8	15"		121.9
16"		157.3	16"		156.6
17"	6.36 (1H, d, *J*=2.5Hz)	104.2	17"	6.50 (d, *J*=2Hz)	103.5
18"		157	18"		157.9
19"	6.46 (dd, *J*=8.5Hz)	110.5	19"	6.30 (dd, *J*=8.4Hz, 2.3Hz)	107.5
20"	7.00 (d, *J*=9Hz)	126.3	20"	7.00 (d, *J*=8Hz)	128.7
21"	2.99-2.95 (m)	23.2	21"	3.25 (d, *J*=7.5Hz)	22.2
22"	5.16 (m)	124.1	22"	5.14 (t, *J*=7.25Hz)	123.1
23"		131.9	23"		131.5
24"	1.80 (s)	25.9	24"	1.70 (s)	25.8
25"	1.56 (s)	17.9	25"	1.56 (s)	17.8

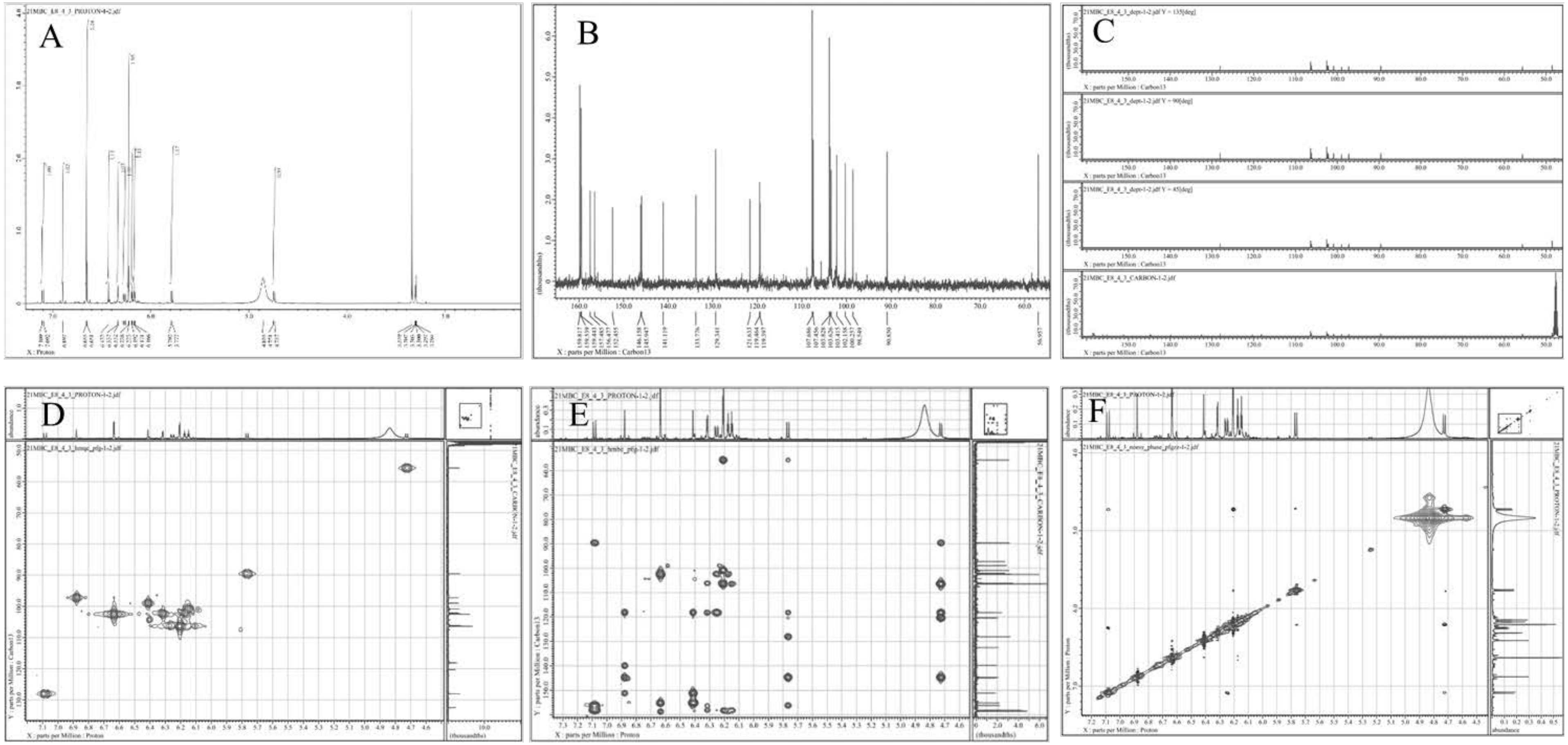

Figure S1. The NMR chromatograms of compounds 4

A: ^{1}H NMR; B: ^{13}C NMR; C: DEPT; D: HMQC; E: HMBC; F: NOESY (dissolved in MeOH-d_4)

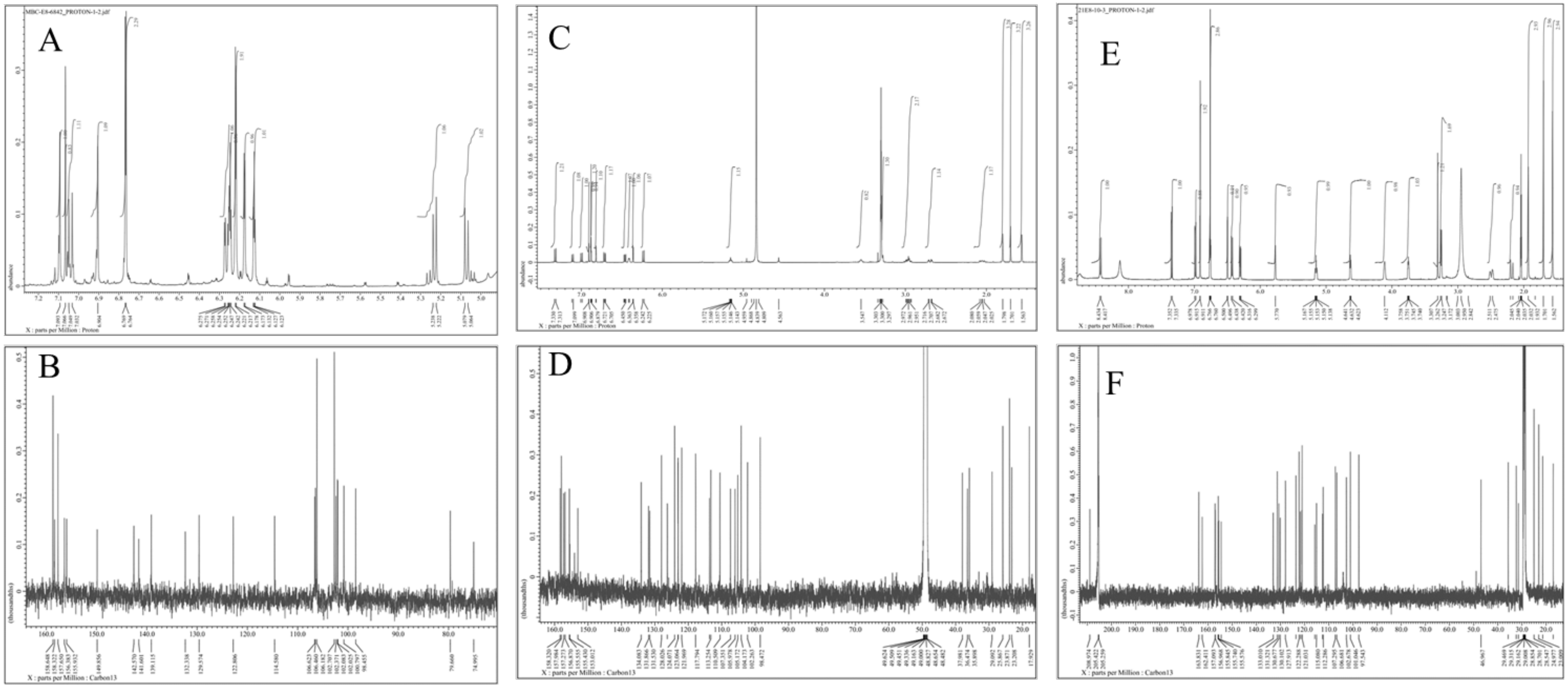

Figure S2. The NMR chromatograms of compounds 6, 7, and 8

A: ^{1}H NMR of compound **6**; B: ^{13}C NMR of compound **6**; C: ^{1}H NMR of compound **7**; D: ^{13}C NMR of compound **7**; E: ^{1}H NMR of compound **8**; F: ^{13}C NMR of compound **8**; (dissolved in MeOH-d_4 and Acetone-d_6)

Table S4. The 1D NMR data of compounds 1- 3

p-Coumaric acid (**1**)
^{1}H NMR: (500 MHz, MeOH-d_4) δ_H 7.52 (d, *J* = 16Hz, 1H, H-7), 7.41 (d, *J* = 8.5Hz, 2H, H-2, 6), 6.77 (d, *J* = 8.5Hz, 2H, H-3, 5), 6.29 (d, *J* = 16Hz, 1H, H-8); ^{13}C NMR: (125 MHz, MeOH-d_4) δ_C 172.5 (C-9), 160.7 (C-4), 145.2 (C-7), 130.8 (C-2, 6), 127.2 (C-1), 117.6 (C-8), 116.7 (C-3, 5).
Chlorogenic acid methyl ester (**2**)
^{1}H NMR: (500 MHz, MeOH-d_4) δ_H 7.51 (d, *J* = 15.5Hz, 1H, H-7′), 7.03 (d, *J*=1.9Hz, 1H, H-2′), 6.94 (dd, *J* = 10.3Hz, 1H, H-6′), 6.77 (d, *J* = 8Hz, 1H, H-5′), 6.21 (d, *J* = 16Hz, 1H, H-8′), 5.27 (dd, *J* = 11.3Hz, 1H, H-3), 4.12 (br d, 1H, H-5), 3.72 (dd, *J* = 7.6Hz, 1H, H-4), 3.68 (s, 3H, $COOCH_3$), 2.20 (dd, *J* = 13.5Hz, 1H, H-2a), 2.16 (m, 2H, H-2b, 6a), 2.00 (br d, *J* = 13.5Hz, 1H, H-6b); ^{13}C NMR: (125 MHz, MeOH-d_4) δ_C 174.1 (C-7), 167 (C-9′), 148.4 (C-4′), 145.9 (C-7′), 145.5 (C-3′), 126.3 (C-1′), 121.6 (C-6′), 115.2 (C-5′), 113.8 (C-2′), 113.7 (C-8′), 74.5 (C-1), 71.2 (C-4), 70.8 (C-3), 69 (C-5), 51.6 (OCH_3), 36.7 (C-6a, 6b), 36.4 (C-2a, 2b).
Oxyresveratrol (**3**)
^{1}H NMR (500 MHz, MeOH-d_4) δ_H 7.32 (d, *J* = 9Hz, 1H, H-6), 7.26 (d, *J* = 16Hz, 1H, H-β), 6.80 (d, *J* = 16.5Hz, 1H, H-α), 6.44 (d, *J* = 2Hz, 2H, H-2′, 6′), 6.31-6.29 (m, 2H, H-3, 5), 6.13 (t, *J* = 2Hz, 1H, H-4′); ^{13}C NMR: (125 MHz, MeOH-d_4) δ_C 158.2 (C-5′, 3′), 157.9 (C-4), 156 (C-2), 140.9 (C-1′) 127.1 (C-6), 125.3 (C-β), 123.6 (C-α), 116.5 (C-1), 107.1 (C-5), 104.3 (C-2′, 6′), 102.2 (C-3), 101 (C-4′).
